# Shifting perspectives: an investigation of a virtual reality awe experience in people going through cancer treatment

**DOI:** 10.1007/s00520-025-09192-4

**Published:** 2025-02-01

**Authors:** V. Tao, G. Tennant, C. Clayden, Lisa M. Reynolds

**Affiliations:** 1 Department of Psychological Medicine, Faculty of Medical and Health Sciences, University of Auckland, 22-30 Park Avenue, Grafton, Auckland, 1023 New Zealand; 2 Harbour Cancer & Wellness, Southern Cross North Harbour Hospital, 212 Wairau Road, Wairau Valley, Auckland, 0627 New Zealand

**Keywords:** VR, Cancer, Awe, Nature

## Abstract

**Purpose:**

Evoking awe using virtual reality appears to be a promising intervention that has potential to positively impact physical and psychological well-being. The purpose of this exploratory study was to investigate the acceptability and potential benefits of a VR experience of awe for patients undergoing cancer treatment.

**Methods:**

Twenty cancer patients viewed a 5-min VR nature experience designed to induce awe and completed questionnaires assessing anxiety, symptom distress, spirituality, and connectedness to nature. Qualitative interviews assessed acceptability and ways to improve the intervention.

**Results:**

The VR experience effectively induced awe. Following the experience, participants reported decreased anxiety and symptom distress and increased feelings of spirituality and connectedness to nature. Participants enjoyed the experience and said they would use it again and would recommend it to others.

**Conclusion:**

VR nature interventions that induce awe are worthy of future investigation as a psychological approach to support cancer patients undergoing treatment.

## Introduction

Coping with cancer and its treatments can be both physically [1], and psychologically challenging [2]. Patients often cope with such challenges by shutting out the outside world and temporarily living in a “cancer bubble” [3], withdrawing themselves from other areas of life [3]. With respect to the inherent challenges that accompany cancer, this withdrawal strategy risks patients losing perspective and their place in the world. Aiming to target cancer-related distress, traditional pharmacological and psychological treatments can be lengthy, resource intensive, and may not directly address this narrowed shift in perspective.

Awe is a complex emotion characterised by “the feeling of being in the presence of something vast that transcends your understanding of the world” [4]. Awe can be elicited in social, spiritual, and physical contexts [4]. However, arguably, the most potent source of awe is in response to nature views such as panoramas of mountains, forests, canyons, and oceans [5, 6]. It has been described as having a self-transcendent quality with the potential of deep, radical, and enduring transformative experiences [7]. Studies in non-cancer groups have shown that awe experiences produce positive changes to nervous system activity [8], increased connectedness [9], an enhanced sense of purpose [10], and improvements to mood and well-being [11]. Although untested in cancer populations, social, cognitive, physical, and psychological benefits of awe may support patients undergoing cancer treatment.

Common awe induction methods such as using recall [12], static images [13], and videos [14] have lacked the power to induce a high-intensity experience of awe [13]. Virtual reality (VR) is a promising tool that offers an intensity of experience that other mediums cannot generally match. VR provides an immersive 3D interface that delivers a computer-generated virtual experience that resembles real-world environments [15]. The VR experience involves a head-mounted display with built-in headphones, drawing the user’s attention away from their immediate surroundings and in to a virtual world [7]. A pioneering study testing the potential of VR-induced awe concluded that an immersive VR video induced more intense feelings of awe and a higher sense of engagement compared to traditional 2D videos [16]. Subsequently, researchers have confirmed that nature-based virtual environments such as forests and mountain summits are more awe-inducing than other stimuli [17].

VR has emerged as a popular healthcare intervention in various clinical settings, including symptom management for patients during cancer treatment [18], and has demonstrated positive effects on physical and psychological symptoms [19]. Despite the progressing evidence using VR as a tool to induce awe, cancer patients have been overlooked in this area. Given the physical and psychological challenges associated with cancer treatment and the potential benefits of experiencing awe, it seems likely that a VR intervention that induces awe might offer benefit to a cancer population undergoing treatment. Here, we report an exploratory study investigating the acceptability and potential benefits of a VR intervention designed to induce awe in patients undergoing cancer treatment.

## Materials and methods

This study utilised a within-subjects mixed methods design wherein all participants received the VR experience. Outcomes were measured pre- and post-intervention with a follow-up phone call approximately 3 days after receiving the intervention. Ethical approval was granted by the Auckland Health Research Ethics Committee (ref: AH24304). All participants gave informed consent to participate.

### Participants

Patients undergoing cancer treatment were invited to participate through cancer support group networks and flyers posted at cancer clinics, support organisations, and online via appropriate community pages. Given the exploratory nature of this study, a sample of twenty participants was deemed sufficient to assess the acceptability and potential benefits of the VR intervention. Participants were required to have a diagnosis of cancer (any type, any stage), be currently undergoing cancer treatment, be over 18 years, and to be able to physically wear the VR headset for 5 min. Participants were excluded if they had a visual, hearing, or cognitive impairment that would limit their ability to take part in the study, or if they could not read, speak, or write in English. Participants received a NZD$20 voucher for their participation.

### Outcome measures

Participants completed written questionnaires at baseline (T1) and post-intervention (T2).

Demographic information collected included age, gender, ethnicity, relationship status, and employment status. Clinical information included type of cancer, stage, time of first diagnosis, previous treatments, and current treatments. Participants’ VR usage history was also collected.

The Functional Assessment of Chronic Illness Therapy Spiritual Well-being Scale (FACIT-Sp-12; [20]) measures spiritual well-being across three domains: peace, meaning, and faith. Participants rate 12 statements such as “I feel peaceful” as it applies to right now on a five-point Likert scale from 0 (*not at all*) to 4 (*very much*). Scores were summed with higher scores indicating a high spiritual well-being state. The scale demonstrated acceptable reliability in the current study (T1 *a* = 0.67; T2 *a* = 0.70).

State anxiety was measured using the six-item short form of the Spielberger State-Trait Anxiety Inventory (STAI-6) [21]. Participants rated six statements describing feelings of calm, tense, upset, relaxed, content, and worried as it applies to right now on a 4-point scale ranging from 1 (*not at all*) to 4 (*very much*). Scores were summed with higher scores indicating greater state anxiety. In the current study, there was good internal reliability at T1 (*a* = 0.79) and T2 (*a* = 0.78).

The Memorial Symptom Assessment Scale, Short Form (MSAS-SF) [22] measured the distress associated with 32 commonly experienced symptoms associated with cancer treatment. Physical symptoms such as “pain, lack of energy and nausea” and psychological symptoms such as “sadness, worry and nervousness” were included. The questionnaire was adapted to ask participants about their experience “right now” and rated on a 5-point scale from 1 (*not at all*) to 5 (*very much*), with a value of 0 being assigned if a symptom was not present. Mean scores were calculated with higher scores indicating greater distress. There was excellent internal reliability (*a* = 0.95) at both timepoints in the current study.

Connectedness to nature was measured using a single-item graphic measure. The Inclusion of Nature in the Self (INS) [23] assesses the extent to which an individual includes nature in their cognitive representation of their self. The scale provides seven images of two circles labelled as “you” and “nature” ranging from two circles being very distant (scored as 1) to almost completely overlapping (scored 7). Participants select the image that best represents their relationship with nature as it applies right now, with a higher score indicating a greater connection to nature. The INS has demonstrated sound content and predictive validity in other work [24].

To assess whether the VR experience successfully induced awe, a 10-item mood induction measure was created in line with previous studies [25]. Participants rated the extent to which they experienced each of ten emotions: anger, disgust, fear, pride, amusement, sadness, joy, awe, anxious, and relaxed on a 7-point scale ranging from 1 (*not at all*) to 7 (*extremely*). The induction of awe was assessed by comparing the change in ratings of awe before and after the VR experience.

The Awe Experience Scale (AWE-S) [26] assessed the experience of awe after the VR experience. Participants rated 30 items across six subscales: alterations in time, self-diminishment, connectedness, perception of vastness, physical sensations, and need for accommodation on a 7-point scale ranging from 1 (*strongly disagree)* to 7 (*strongly agree).* The questionnaire was adapted to be specific to the current VR experience. Scores were summed with higher scores indicating that the experience was more awe-inspiring. The scale has demonstrated strong internal consistency (*a* = 0.92) and has established convergent and divergent validity [26]. The scale had excellent internal reliability in the current work (*a* = 0.91).

To gain a deeper understanding of participants experience and feedback about how to improve the VR experience, participants were asked open-ended questions via follow-up phone call focussing on five areas: overall experience, likes, dislikes, acceptability, and recommendations.

### VR experience

The immersive VR experience used in this study was created by Galaxiid (https://www.galaxiid.com/). The 5-min 360° video was compiled of New Zealand nature scenes specifically designed to induce a feeling of awe (see YouTube: https://www.youtube.com/watch?v=HieBPYFmEBg). There was a total of eight awe-inspiring scenes (Table 1) accompanied by a meditative music track (Fields of Meditation by Emmanual Jacob) and the natural sounds associated with each scene such as bird song, leaves rustling in the wind, waves lapping, and water falling.

**Table 1:**
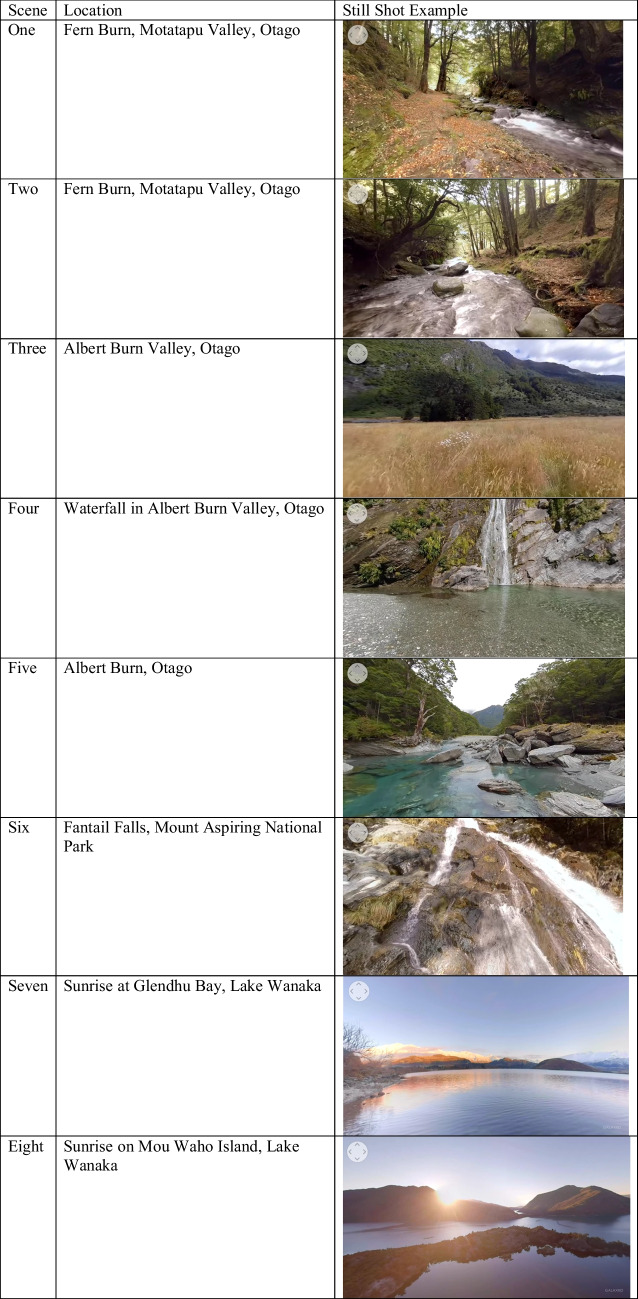
Location and still shot of each scene

### Procedure

Participants began by completing baseline written questionnaires. This was followed by a brief 2D video where a researcher (LR) described awe and encouraged participants to be aware of moments of awe when viewing the VR experience. Participants were then fitted with the VR headset and viewed a 5-min immersive VR experience. Participants completed the post-intervention written questionnaires. Approximately 3 days after their VR experience, participants received a follow-up phone call to gather their feedback.

### Statistical analyses

Data were analysed using SPSS version 26. Normality tests were conducted and revealed highly skewed data; therefore, non-parametric testing was required. Wilcoxon signed-rank tests were performed to analyse changes in outcome measures before and after the VR experience. A *p*-value of < 0.05 was considered statistically significant in this study. Qualitative data were analysed using thematic analysis [27].

## Results

A total of 27 individuals expressed interest and 20 of these met the inclusion criteria and agreed to participate. No participants were lost to follow-up. The mean age of the sample was 64 years, there was an adequate split across genders (male = 45%), and ethnicity was primarily NZ European (70%, *n* = 14). Most participants had never used VR before (80%, *n* = 16). There was a range of various cancer types and stages, with most participants being first diagnosed less than 1 year ago (70%, *n* = 14). Some participants were undergoing multiple treatments simultaneously (see Table 2).

**Table 2:** Demographic and clinical characteristics of the sample

Measure		Mean (*SD*) or *N* (%)
		All participants (*N* = 20)
Age		64.75 (11.53)
Gender	Male	9 (45%)
	Female	11 (55%)
Ethnicity	NZ European	14 (70%)
	Māori	2 (10%)
	Chinese	2 (10%)
	Other	2 (10%)
Relationship status	Single	2 (10%)
	In a relationship	3 (15%)
	Married	14 (70%)
	Widowed	1 (5%)
Employment status	Full-time	9 (45%)
	Part-time	1 (5%)
	Contract/casual	2 (10%)
	Homemaker	1 (5%)
	Retired	7 (35%
Cancer type	Breast	5 (25%)
	Prostate	8 (40%)
	Lung	1 (5%)
	Colorectal	1 (5%)
	Ovarian	2 (10%)
	Skin	1 (5%)
	Brain/nervous system	1 (5%)
	Head and neck	1 (5%)
Cancer stage	1	3 (15%)
	2	4 (20%)
	3	2 (10%)
	4	6 (30%
	Other/I don’t know	5 (25%)
First diagnosed	< 1 year	14 (70%)
	2 years ago	1 (5%)
	3 years ago	1 (5%)
	5 years ago	1 (5%)
	9 years ago	1 (5%)
	> 10 years ago	2 (10%)
Current cancer treatment*	Chemotherapy	6 (30%)
	Radiation therapy	15 (75%)
	Immunotherapy	2 (10%)
	Hormone therapy	2 (10%)
	Surgery	3 (15%)
Past VR use	Never	16 (80%)
	Once or twice	4 (20%)

### Did the VR experience induce awe?

**Fig. 1:**
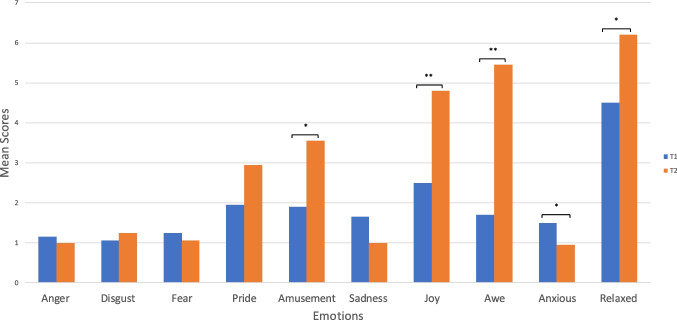
Bar graph illustrating the mean scores of ten different emotions before and after the VR experience

There was an increase in ratings of awe after the VR experience (*M* = 5.45, *SD* = 1.64) compared to before the experience (*M* = 1.70, *SD* = 1.30, *Z* = 3.81, *p* < 0.001). There were also increases in amusement (*Z* = 2.66, *p* = 0.007), joy (*Z* = 3.31, *p* < 0.001), relaxation (*Z* = 2.72, *p* = 0.006), and a decrease in feeling anxious (*Z* = 2.04, *p* = 0.041); however, the effect size for changes in awe was largest (see Fig. 1).

**Fig. 2:**
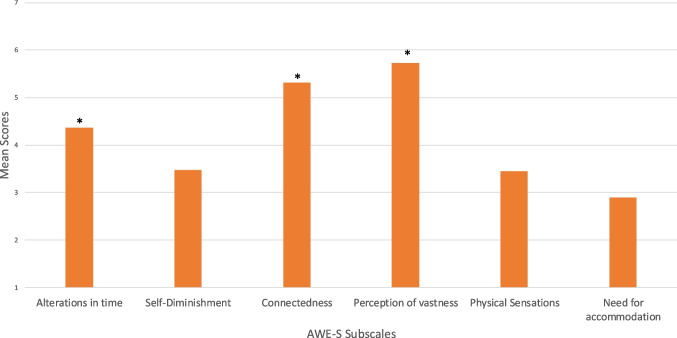
Bar graph illustrating the mean scores of the six subscales in the AWE-S

The AWE-S was administered post-VR experience. The mean scores for each subscale were calculated, revealing that three facets of awe were rated as above a neutral score of 4 following the VR experience: “alterations in time” (*M* = 4.37), “connectedness” (*M* = 5.32), and a “perception of vastness” (*M* = 5.73) (see Fig. 2).

Many participants gave unprompted feedback expressing their feelings of awe during the VR experience: *“definitely put me in a feeling of awe.”*

### Did the VR experience effect outcomes?

#### Anxiety

**Fig. 3:**
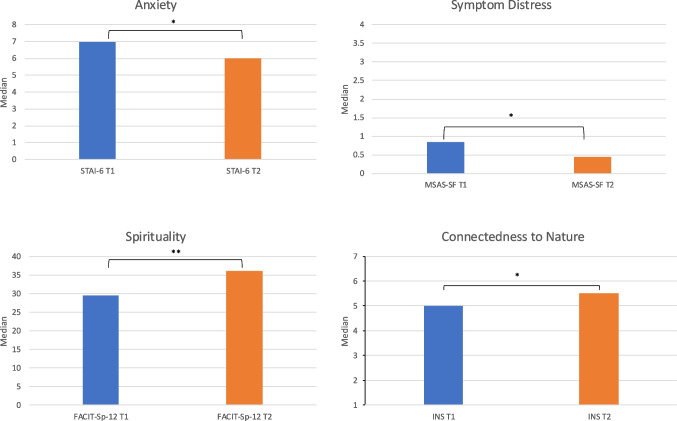
Bar graph comparing the outcomes before and after the VR experience. *p < 0.050; **p < *0.001*

STAI-6 scores significantly decreased after the VR experience (*Z* = 2.20, *p* = 0.028.) (see Table 3, Fig. 3). Participants also voiced feelings of being more relaxed and calm after the VR experience in their follow-up interviews, reflected in statements like the following:

*“I felt braver. I felt more confident within myself … I wish it was available for people to do especially in those low times because it really really does lift your spirits and makes you feel braver.”*

**Table 3:** Psychometric properties of the outcomes before and after the VR experience

Scale	Mdn	IQR	Range
STAI-A-6 T1	7.00	10.00	6–16
STAI-A-6 T2	6.00	6.00	6–12
MSAS-SF T1	0.84	3.13	0.06–3.19
MSAS-SF T2	0.45	2.41	0.06–2.47
FACIT-Sp T1	29.50	26.00	22–48
FACIT-Sp T2	36.00	25.00	23–48
INS T1	5.00	5.00	2–7
INS T2	5.50	4.00	3–7

#### Symptom distress

MSAS-SF scores also significantly decreased following the VR experience compared to baseline (*Z* = 2.99, *p* = 0.003). Again, participant feedback supported this finding with one participant reporting that the VR experience was helpful in coping with the physical aspects of treatment beyond simply distraction:

*“I felt I was able to eat which I hadn’t done for two weeks. I was actually quite hungry, which is very positive for me, and I was able to actually have something to eat. It was a huge step for me.”*

#### Spirituality

There was a significant increase in FACIT-Sp-12 scores after the VR experience (*Z* = 3.73, *p* < 0.001). Whilst spirituality means different things to different people, one participant described the VR experience as strengthening their relationship with God:

*“Made my belief in God even stronger than what it was.”*

#### Connection to nature

Finally, there was also a significant increase in INS scores following the experience (*Z* = 2.66, *p* = 0.008). Participant feedback mirrored this finding, commenting on how it felt they were really in nature and crediting the level of immersion as fostering a more potent, deeper connection to nature:

*“It felt very very real. It felt like I was actually out in nature, which is something I hadn’t really been able to do for the past two months really with the chemo. … I think the VR experience gave me that, like as real as we were … hearing the sounds of the forest, you could almost get a mossy smell.”*

### Acceptability

Feedback about the VR experience was strongly positive and it appeared to be an acceptable intervention to participants:

*“I felt it was incredible. It just made me feel so relaxed. It just was very soothing, very calming, the whole experience. … I think one of the greatest things that I’ve done in a long time.”*

Participants were also very likely to use the VR experience again and recommend it to others undergoing similar treatments:

*“If I could, I’d do one [VR experience] every week.”*

*“I think anybody of any age could do it and enjoy it, get the advantage of it.”*

Participants recognised the VR experience as a potential cancer coping tool to aid in both the physical and emotional aspects of treatment:

*“I thought it was a refreshing approach as an aid towards recovery or coping with what’s happening in my life, so I thought it was quite beneficial. … I made an effort to look back as to what the VR experience was about, so while I was having my treatment, I made sure that I went back and recall[ed] the experience. … I think it’s a well-rounded approach to help people relax and unwind and re-evaluate the important things in life.”*

However, participants also suggested improvements to enhance the experience. Suggestions included having *“a variety of different scenes for different people”*, *“make it longer, about 10 min”*, to *“skip the music”* and to increase the clarity, *“the resolution is too low for me”*. No one reported cyber-sickness during the VR experience; however, a few participants commented on feeling slightly disoriented after returning to reality: *“when I tried to stand up* [after], *it kind of made me feel a bit woozy”*.

## Conclusion

The VR experience used in the current study was designed to induce awe in patients undergoing cancer treatment by exposing them to immersive scenes of nature that were vast and majestic. Our findings revealed that the VR experience effectively induced a feeling of awe and that particular dimensions of awe were strongest including alterations in time, connectedness, and a perception of vastness. The current study extends the literature by demonstrating that VR can induce awe in a population going through cancer treatment. Although the aims of this work were exploratory, the VR experience was nevertheless associated with significant improvements in both physical (symptom distress) and psychological (anxiety, spirituality, connection with nature) outcomes. All outcomes demonstrated medium to large effect sizes.

The fact that anxiety and symptom distress both decreased following the intervention has clinical implications in a population where anxiety and symptom distress are negatively linked to quality of life [28]. Participants viewed the VR experience as a potential tool to cope with their cancer and the physical and emotional impacts of treatment. In practice, this could provide patients with an alternative approach to help ease their anxiety and distress. Whilst pharmacological approaches [29, 30] are a current option, some patients may not want to take further drugs or may have concerns about other side effects [31]. Psychological approaches such as mindfulness-based interventions [32] and Acceptance Commitment Therapy [33] have proven effective, however, can be expensive, lengthy, and require administration by skilled professionals, therefore limiting accessibility [34]. In contrast, VR interventions are accessible and relatively affordable. They can be provided in a timely manner offering a type of therapy that patients can self-engage with. Thus, VR has potential to provide a means of support that teams in both hospital settings and outpatient services could implement.

An increase in feelings of spirituality and connectedness to nature in this study is consistent with research showing awe can lead to stronger spirituality [35] and feelings of nature connectedness [9, 36]. Research has shown that exposure to nature can decrease stress and negative effect [37]; however, access to nature is not always feasible for patients undergoing intensive treatment [1]. The current VR experience offered an accessible way for patients to experience the benefits of nature. This possibility is important given evidence that the benefits of immersion in a real nature setting are comparable to the benefits of virtual exposure to that same setting [38]. Furthermore, increasing feelings of spirituality can be important as cancer patients face existential concerns and questions about life [39]. Connecting with faith and spirituality can offer a source of meaning-making. Feelings of connectedness may also help counter feelings of isolation [3]. This VR intervention appears to be a pragmatic means for cancer patients to connect to what is meaningful to them in a potentially restorative and healing way.

Finally, positive participant feedback suggests that the VR experience was acceptable to this population. Participants said that they were likely to use the intervention again themselves and would recommend it to others. Although previous work has cited VR side effects such as cybersickness [40], there were no reported complaints of discomfort or VR-induced nausea. Although some reports of brief disorientation, this feeling appeared to quickly resolve. Future interventions should allow patients space and time following the VR experience to re-orient themselves to physical reality.

Although this unique exploratory study has strengths, it is not without limitations. The limited sample size of 20 participants lacked diversity and constrains the generalisability of findings to broader populations. Future studies should employ a larger sample size to improve the generalisability of findings to broader cancer patient populations, and to determine whether there are differences across demographics such as age or ethnicity. The small sample size also limits our ability to conduct moderation analyses. It would be useful to know whether the benefits from the intervention are greater for people going through one type of treatment compared to another (e.g. chemotherapy vs. radiotherapy). This would enable the intervention to be targeted towards sub-groups with the most potential for experiencing benefits. Additionally, this intervention consisted of only one brief VR session. Whilst this allowed flexibility and meant it could be delivered in a timely manner, it is unclear whether the benefits of this brief dose might be improved in a longer experience or whether benefits might be sustained over time. Future research could consider a longitudinal approach and include follow-up assessment at various time points after the intervention.

In conclusion, this exploratory study suggests that a VR experience of awe is not only associated with improvements to physical and psychological well-being but is an acceptable intervention for patients undergoing cancer treatment.

## Data Availability

No datasets were generated or analysed during the current study.
